# Pharmacist-Driven Transitions of Care Practice Model for Prescribing Oral Antimicrobials at Hospital Discharge

**DOI:** 10.1001/jamanetworkopen.2022.11331

**Published:** 2022-05-10

**Authors:** Nicholas J. Mercuro, Corey J. Medler, Rachel M. Kenney, Nancy C. MacDonald, Melinda M. Neuhauser, Lauri A. Hicks, Arjun Srinivasan, George Divine, Amy Beaulac, Erin Eriksson, Ronald Kendall, Marilen Martinez, Allison Weinmann, Marcus Zervos, Susan L. Davis

**Affiliations:** 1 Department of Pharmacy, Henry Ford Health System, Detroit, Michigan; 2 Eugene Applebaum College of Pharmacy, Wayne State University, Detroit, Michigan; 3 Department of Pharmacy, Maine Medical Center, Portland; 4 National Center for Emerging and Zoonotic Infectious Diseases, Centers for Disease Control and Prevention, Division of Healthcare Quality Promotion, Atlanta, Georgia; 5 Division of Public Health Sciences, Henry Ford Health System, Detroit, Michigan; 6 Division of Infectious Diseases, Henry Ford Health System, Detroit, Michigan

## Abstract

**Importance:**

Although prescribers face numerous patient-centered challenges during transitions of care (TOC) at hospital discharge, prolonged duration of antimicrobial therapy for common infections remains problematic, and resources are needed for antimicrobial stewardship throughout this period.

**Objective:**

To evaluate a pharmacist-driven intervention designed to improve selection and duration of oral antimicrobial therapy prescribed at hospital discharge for common infections.

**Design, Setting, and Participants:**

This quality improvement study used a nonrandomized stepped-wedge design with 3 study phases from September 1, 2018, to August 31, 2019. Seventeen distinct medicine, surgery, and specialty units from a health system in Southeast Michigan participated, including 1 academic tertiary hospital and 4 community hospitals. Hospitalized adults who had urinary, respiratory, skin and/or soft tissue, and intra-abdominal infections and were prescribed antimicrobials at discharge were included in the analysis. Data were analyzed from February 18, 2020, to February 28, 2022.

**Interventions:**

Clinical pharmacists engaged in a new standard of care for antimicrobial stewardship practices during TOC by identifying patients to be discharged with a prescription for oral antimicrobials and collaborating with primary teams to prescribe optimal therapy. Academic and community hospitals used both antimicrobial stewardship and clinical pharmacists in a multidisciplinary rounding model to discuss, document, and facilitate order entry of the antimicrobial prescription at discharge.

**Main Outcomes and Measures:**

The primary end point was frequency of optimized antimicrobial prescription at discharge. Health system guidelines developed from national guidelines and best practices for short-course therapies were used to evaluate optimal therapy.

**Results:**

A total of 800 patients prescribed oral antimicrobials at hospital discharge were included in the analysis (441 women [55.1%]; mean [SD] age, 66.8 [17.3] years): 400 in the preintervention period and 400 in the postintervention period. The most common diagnoses were pneumonia (264 [33.0%]), upper respiratory tract infection and/or acute exacerbation of chronic obstructive pulmonary disease (214 [26.8%]), and urinary tract infection (203 [25.4%]). Patients in the postintervention group were more likely to have an optimal antimicrobial prescription (time-adjusted generalized estimating equation odds ratio, 5.63 [95% CI, 3.69-8.60]). The absolute increase in optimal prescribing in the postintervention group was consistent in both academic (37.4% [95% CI, 27.5%-46.7%]) and community (43.2% [95% CI, 32.4%-52.8%]) TOC models. There were no differences in clinical resolution or mortality. Fewer severe antimicrobial-related adverse effects (time-adjusted generalized estimating equation odds ratio, 0.40 [95% CI, 0.18-0.88]) were identified in the postintervention (13 [3.2%]) compared with the preintervention (36 [9.0%]) groups.

**Conclusions and Relevance:**

The findings of this quality improvement study suggest that targeted antimicrobial stewardship interventions during TOC were associated with increased optimal, guideline-concordant antimicrobial prescriptions at discharge.

## Key Points

**Question:**

Are antimicrobial stewardship interventions during transitions of care associated with improved prescribing of antimicrobials at hospital discharge?

**Findings:**

In this quality improvement study of 800 patients, a pharmacist-driven intervention targeting antimicrobial prescribing at discharge was associated with higher frequency of optimal antimicrobial regimens compared with before the intervention. Patients in the postintervention group had similar rates of mortality, readmission, and clinical resolution and fewer severe antimicrobial-related adverse effects compared with the preintervention group.

**Meaning:**

These findings suggest that hospitals can leverage resources toward antimicrobial stewardship during transitions of care to optimize antimicrobial therapy.

## Teaser

This nonrandomized, stepped-wedge quality improvement study assesses a pharmacist-led antimicrobial stewardship program to review and identify inappropriate prescriptions at hospital discharge and evaluate the association of the intervention with optimized therapy and adverse events among patients with common infections.

## Introduction

In the US, 1 in 5 hospitalized adults is prescribed an antimicrobial at the time of discharge, accounting for millions of antimicrobial-days each year.^1^ Although prescribers face numerous patient-centered challenges during transitions of care (TOC), resources are sorely needed for antimicrobial stewardship throughout this period.^2^ In a statewide collaborative effort, the Michigan Hospital Medicine Safety Consortium classified the appropriateness of more than 20 000 antimicrobials prescribed at discharge, and nearly half were considered overuse.^3^ The most common antimicrobial class prescribed at discharge, the fluoroquinolone, has a number of box warnings and safety concerns, yet as few as 25% of these orders are considered appropriate.^1,4,5^ Prolonged duration of antimicrobial therapy is consistently the major contributor to inappropriate prescribing during TOC, and a mean of 40% of each patients’ total duration of therapy is administered post discharge.^1,3,6^ The review of antimicrobial prescribing during TOC represents a crucial moment in a patient’s clinical course to ensure safe, effective, and guideline-concordant therapy.

Few antimicrobial stewardship interventions have been targeted in the collaborative discharge planning process.^7,8,9^ Traditional inpatient antimicrobial stewardship initiatives such as audit and feedback may not impact prescribing practices at discharge.^10,11^ The Centers for Disease Control and Prevention core elements of outpatient antibiotic stewardship^12^ identify transition from acute care to other health care settings as an opportunity to improve the quality of prescribing. Physicians, pharmacists, nurses, and case managers at the front lines of patient care have the tools to collaborate and optimize antimicrobial therapy at discharge.^9^ The purpose of this study was to (1) implement a pharmacist-led, multidisciplinary review of discharge planning for oral antimicrobial therapy; (2) quantify inappropriate antimicrobial prescribing at the time of discharge; and (3) evaluate the association of the intervention with optimized antimicrobial therapy, infection-related readmissions, and antimicrobial-associated harms.

## Methods

### Study Setting and Design

This quality improvement study used a nonrandomized stepped-wedge design to evaluate an antimicrobial stewardship intervention for adults discharged from the hospital with antimicrobial prescriptions for select uncomplicated infections. From September 1, 2018, to August 31, 2019, 5 hospitals within the Henry Ford Health System in southeastern Michigan participated in this study, including Henry Ford Hospital (a 877-bed academic medical center in Detroit), Henry Ford Allegiance Hospital (a 475-bed community hospital in Jackson), Henry Ford Wyandotte Hospital (a 401-bed community hospital in Wyandotte), Henry Ford Macomb Hospital (a 361-bed community hospital in Clinton Township), and Henry Ford West Bloomfield Hospital (a 191-bed community hospital in West Bloomfield). Each hospital had at least a partial full-time equivalent for an antimicrobial stewardship pharmacist (0.8-1.0 full-time equivalent) and physician (0.2-0.8 full-time equivalent), and clinical pharmacists were integrated within medical teams. The intervention was implemented across all sites in a nonrandomized order. This intervention was selected for feasibility of implementing a new standard of care at TOC with the available resources in the health system. The stepped-wedge design also allowed control for regression to the mean, maturation effects, and confounding due to secular trends in a health system–wide intervention that implemented the TOC initiative for 3 phases (Table 1) that were selected in order of institutional readiness.^13^ The study was approved by the health system’s ethics committee as a quality improvement initiative, and a waiver of informed consent was granted by the institutional review board. This study followed the Standards for Quality Improvement Reporting Excellence (SQUIRE 2.0) reporting guideline.

**Table 1.** Description of Service Teams in Study Groups and Timeline of Nonrandomized, Stepped-Wedge Design and Interventions

Study group	Quarter 1: August to October 2018 (n = 125)	Quarter 2: November 2018 to February 2019 (n = 300)	Quarter 3: March to May 2019 (n = 225)	Quarter 4: June to August 2019 (n = 150)
Group 1 (n = 250): academic hospital service teams including internal medicine, pulmonology, family medicine, and infectious diseases	Preintervention	Intervention in place	Intervention in place	Intervention in place
Group 2 (n = 275): academic and community hospital service teams including nephrology, cardiology, family medicine, and internal medicine	Preintervention	Preintervention	Intervention in place	Intervention in place
Group 3 (n = 275): community service teams including hospitalist and internal medicine	Preintervention	Preintervention	Preintervention	Intervention in place

### Intervention

The objective of the intervention was to facilitate optimal antimicrobial discharge prescriptions by leveraging the existing pharmacy practice model for TOC with local antimicrobial use and duration guidelines through collaboration with the primary team. The antimicrobial stewardship intervention at TOC was implemented in 3 phases in 17 distinct units (service teams) across the 5 hospitals. Order of intervention rollout was prioritized based on patient volume, availability of resources, and pharmacist training. Group 1 consisted of units at an academic hospital; group 2, both academic and community hospital units; and group 3, community hospital units (Table 1). The TOC model enabled clinical and antimicrobial stewardship pharmacists to identify patients approaching discharge with active antimicrobial orders, to create and communicate collaborative plans related to antimicrobial selection and duration of therapy, and to enter the antimicrobial prescription with a stop date to be signed by the primary clinician at discharge. Local physician champions were identified on each service team to promote intervention uptake.^14^

Clinical pharmacists involved with the intervention were trained in mandatory competency sessions to optimally manage workflow and the operational components for antimicrobial order entry. Before discharge, the pharmacist reviewed an inpatient team census in the electronic health record that included all active antimicrobials at the start of each shift. Patients were reviewed to identify those with qualifying diagnoses of infectious diseases who may be eligible to complete the antimicrobial course with oral therapy after discharge. In the academic center setting, clinical pharmacists assessed discharge readiness with a notification in the electronic team census in addition to daily discussions on collaborative rounds with nurses, physicians, nurse practitioners, physician assistants, and case managers. To identify patients with anticipated discharge in community hospital settings, antimicrobial stewardship pharmacists were alerted in different ways depending on reporting structures specific to each institution. These methods included electronic team census notifications, direct communication from nursing and case management, and/or discussions during collaborative team rounds. Cost inquiries for oral antimicrobials were requested on a case-by-case basis via electronic order to the outpatient pharmacist to address financial barriers.^15^ Documentation in the electronic medical record was completed by the clinical pharmacist to describe the agent, indication, dose, and duration of therapy for patients during the study period. The discharging prescriber received recommendations for the protocolized antimicrobial regimen during TOC on collaborative rounds or via telephone. After the antimicrobial plan was discussed, the orders for discharge were entered or modified (if needed) in the electronic discharge queue by the pharmacist to be cosigned by the prescriber. Protocol adherence was monitored by assessing documentation in the medical record of intervention completion, and progress was communicated to clinical pharmacists and physician champions each month via internal posters, meetings, and email. To increase intervention uptake, physician champions introduced practice model changes during departmental meetings, signed a letter in support of the intervention, and shared their photographs on a poster for monitoring protocol adherence.

### Study Population

Adults admitted to general medical and/or surgical wards who were discharged with oral antimicrobial therapy were eligible for inclusion. Patients who were pregnant, discharged with parenteral antimicrobials, or diagnosed with cystic fibrosis, endovascular infections, central nervous system infections, osteomyelitis, or febrile neutropenia were excluded. Diagnoses of interest included common infections with evidence-based guideline recommendations for antimicrobial courses (eMethods in the Supplement): infections of the urinary tract, respiratory tract, skin and/or skin-structure sites, and intra-abdominal sites with adequate source control (eFigure 1 in the Supplement).^16^ Study participants were identified among those patients discharged with a prescription for oral antimicrobials from the data repository of the electronic medical record. Cases were sorted and selected using a random number generator (Excel, version 15.0 [Microsoft Corporation]) and screened until 25 patients in each group every month were included. Electronic medical records were manually reviewed to ascertain data and then entered in REDCap (Research Electronic Data Capture). Patient race and ethnicity data were collected using the demographic populated fields in the electronic medical record and reported to identify possible differences between groups.

### Patient Data and End Point Definitions

The primary end point was frequency of discharge with an optimized antimicrobial regimen, determined by review of medical records, prescriptions, and discharge. Health system guidelines were used to assess appropriateness of antimicrobial selection, dose, and duration. Definitions for optimal antimicrobial therapy were modified in alignment with those proposed by Spivak et al^17^ (eMethods in the Supplement). Hospital length of stay and antimicrobial duration of therapy were assessed as resource use outcomes. Safety end points included antimicrobial-related adverse effects (ADEs), 30-day unplanned office and/or emergency department visits, 30-day readmissions, and 30- and 90-day mortality. Antimicrobial-related ADEs were categorized as mild to moderate or as severe. Severe ADEs that were assessed to 90 days included *Clostridiodies difficile* infection and isolation (from any clinical culture) of a new multidrug-resistant organism,^18^ whereas anaphylaxis and/or angioedema, kidney failure, acute hepatic failure, torsades de pointes, seizure, and serious hematologic toxic effects were measured to 30 days. Mild to moderate ADEs such as diarrhea; QTc prolongation; rash; mild elevations in levels of aminotransferases, bilirubin, and/or creatinine; and others outlined by Tamma et al^19^ were assessed to 30 days. When available, outside electronic health records were ascertained for events that were presumed to not occur if not documented. Clinical resolution was assessed only in patients with available follow-up data, defined as resolution of signs and symptoms such that no further antimicrobial therapy was required after completion of planned therapy for the same indication, to 30 days.^20^

### Statistical Analysis

Data were analyzed from February 18, 2020, to February 28, 2022. SPSS, version 26.0 (IBM Corp), and SAS, version 9.4 (SAS Institute Inc), were used for calculations. Sample size was estimated by presuming a 20% relative reduction of nonoptimized antimicrobial therapy at discharge from 60% to 48% (historic data indicate 54%-63% of patients receiving antimicrobials receive excessive durations^4^). Very roughly approximating the generalized estimating equation (GEE) logistic regression sample size by that for a χ^2^ comparison of proportions, 357 patients in each study arm were needed for a 2-sided α = .05 with 90% power. The Mann-Whitney test was used for nonparametric data and an unpaired 2-tailed *t* test was used for parametric data. We used the χ^2^ and Fisher exact tests for categorical variables, as appropriate. Two-sided *P* < .05 and 95% CIs were used to describe statistical significance. To account for correlation in data from patients treated at the same location, the primary inferential analyses used GEE logistic regression (SAS procedure PROC_GENMOD) and analysis of covariance models, with service team location at discharge used to define clusters. To adjust for potential temporal trend, time in months since the beginning of the study period was used as a covariate in primary analyses. Multivariable, time-adjusted GEE logistic regression was used to identify independent associations with an optimized antimicrobial regimen at discharge. Candidate variables for the multivariable model included age, sex, study month, and select covariates with predetermined clinical suspicion for optimal prescribing. The final model used covariates with *P* < .10 in time-adjusted GEE analysis.

## Results

Of 1440 patients screened, 800 were included across the 3 study phases: 400 in the preintervention period and 400 in the postintervention period (Table 1 and eFigure 2 in the Supplement). The most common reasons for exclusion were at least 1 of the following: complicated or severe infection (n = 423), solid organ transplant or neutropenia (n = 102), transfer to or from an outside hospital or hospice (n = 96), and discharge with intravenous antimicrobial therapy (n = 47) (eFigure 2 and eMethods in the Supplement). A total of 441 included patients (55.1%) were women and 359 (44.9%) were men. The mean (SD) age was 66.8 (17.3) years; mean (SD) body mass index (calculated as weight in kilograms divided by height in meters squared), 29.9 (9.1). The median length of stay was 3 (IQR, 2-5) days. Most patients (427 [53.4%]) were admitted to the academic medical center and discharged home (673 [84.1%]). During the study period, more than 1500 interventions were documented, and overall protocol adherence throughout the health system was 63%. Service teams included medicine, surgery, hospitalist, pulmonology, infectious disease, family medicine, cardiology, and nephrology. The most common diagnoses were pneumonia (264 [33.0%]), upper respiratory tract infection and/or acute exacerbation of chronic obstructive pulmonary disease (214 [26.8%]), urinary tract infection (203 [25.4%]), and skin or soft tissue infection (125 [15.6%]). The median Charlson Comorbidity Index score was 2 (IQR, 1-3); there were no significant differences in comorbid conditions, severity of illness on presentation, or risk factors for multidrug-resistant organisms between groups (Table 2 and eTable 1 in the Supplement).

**Table 2.** Patient Demographic and Clinical Characteristics

Characteristic	Patient group^a^		Time-adjusted GEE *P* value
	Preintervention (n = 400)	Postintervention (n = 400)	
Age, mean (SD), y	69.0 (17.1)	64.5 (17.2)	.67
Sex			
Women	221 (55.3)	220 (55.0)	.60
Men	179 (44.7)	180 (45.0)	
Race and ethnicity			
Black	113 (28.3)	200 (50.0)	.04
White	259 (64.7)	161 (40.3)	
Other or unknown^b^	28 (7.0)	39 (9.7)	
Charlson Comorbidity Index score, median (IQR)	2 (1-3)	2 (1-3)	.96
≥2 SIRS criteria on day 3	22 (5.5)	18 (4.5)	.54
Length of stay, mean (SD), d	3.6 (2.2)	3.3 (2.2)	.70
Any MDRO risk factor	216 (54.0)	210 (52.5)	.12
Admitted in last 90 d	130 (32.5)	117 (29.3)	.99
Antimicrobial therapy in last 90 d	144 (36.0)	154 (38.5)	.65
Prior MDRO colonization	22 (5.5)	27 (6.7)	.48
Immunocompromised	7 (1.7)	15 (3.7)	.04
Nonambulatory status	32 (8.0)	19 (4.7)	.51
Pneumonia	144 (36.0)	120 (30.0)	.24
Community-acquired without risk factors for MDRO	108 (27.0)	92 (23.0)	.03
Community-acquired with risk factors for MDRO	33 (8.3)	24 (6.0)	.45
Hospital-associated	3 (0.7)	4 (1.0)	.60
Acute exacerbation of COPD or upper respiratory tract infection	101 (25.3)	113 (28.3)	.49
Urinary tract infection	115 (28.7)	88 (22.0)	.23
Pyelonephritis	25 (6.3)	23 (5.7)	.91
Complicated urinary tract infection	46 (11.5)	39 (9.7)	.91
Cystitis	44 (11.0)	26 (6.5)	.14
Skin and/or soft tissue infection	53 (13.3)	72 (18.0)	.90
Purulent	20 (5.0)	39 (9.7)	.17
Nonpurulent	33 (8.3)	33 (8.3)	.15
Intra-abdominal infection	4 (1.0)	12 (3.0)	.69

Abbreviations: COPD, chronic obstructive pulmonary disease; GEE, generalized estimating equation; MDRO, multidrug-resistant organism; SIRS, systemic inflammatory response syndrome.

^a^ Unless otherwise indicated, data are expressed as number (%) of patients. Percentages have been rounded and may not total 100.

^b^ Includes race or ethnicity reported as Asian, Pacific Islander, unknown, other, and decline.

The primary end point, optimal antimicrobial prescription at discharge, was associated with intervention implementation (144 of 400 [36.0%] vs 326 of 400 [81.5%]; *P* < .001) (Table 3) and remained consistently associated with improved prescribing across all study phases (eTable 2 in the Supplement). The absolute increase in optimal prescribing in the postintervention group was consistent in both academic (37.4% [95% CI, 27.5%-46.7%]) and community (43.2% [95% CI, 32.4%-52.8%]) hospital models. Patients in the postintervention group were more likely to have an optimal antimicrobial prescription (time-adjusted GEE odds ratio [OR], 5.63 [95% CI, 3.69-8.60]). Reductions in prolonged durations of therapy (177 of 400 [44.2%] vs 37 of 400 [9.2%]; mean difference, −35.0% [95% CI, −40.2% to −29.2%]), non–guideline-concordant antimicrobial selection (81 of 400 [20.2%] vs 24 of 400 [6.0%]; mean difference, −14.3% [95% CI, −18.8% to −9.6%]), and treatment of asymptomatic bacteriuria (37 of 400 [9.2%] vs 10 of 400 [2.5%]; mean difference, −6.8% [95% CI, −10.0% to −3.4%]) were the largest contributing components of improved optimized discharge prescription (Table 3). The intervention was associated with decreased total antimicrobial duration (time-adjusted absolute difference, −1.1 [95% CI, −1.7 to −0.6] antibiotic days) (eTable 3 in the Supplement). Duration of antimicrobial therapy for respiratory tract infection was reduced (time-adjusted absolute difference, −1.8 [95% CI, −2.3 to −1.2] antibiotic-days) in the postintervention period, whereas there was no difference for urinary tract infection or skin and/or soft tissue infections. There were no differences in unadjusted analyses for clinical resolution, readmission at 30 days, or mortality (Table 4). The intervention was associated with fewer ADEs, mostly owing to reductions in more severe ADEs such as a new multidrug-resistant organism and *C difficile* infection (Table 4) by day 90 (severe ADEs, 36 [9.0%] vs 13 [3.2%]; time-adjusted GEE OR, 0.40 [95% CI, 0.18-0.88]).

**Table 3.** Patients Receiving Optimal Prescription at Discharge

Prescription component	Patient group, No./total No. (%)		Absolute difference, % (95% CI)	Time-adjusted GEE OR (95% CI)
	Preintervention	Postintervention		
Overall	144/400 (36.0)	326/400 (81.5)	45.5 (39.2 to51.3)	5.63 (3.69 to 8.60)
Group 1	14/25 (56.0)	185/225 (82.2)	26.2 (7.0 to 45.8)	1.09 (0.59 to 2.01)
Group 2	59/150 (39.3)	103/125 (82.4)	43.1 (32.2 to 52.7)	3.93 (1.72 to 8.99)
Group 3	71/225 (31.6)	38/50 (76.0)	44.4 (30.0 to 56.5)	5.53 (1.59 to 19.23)
Community hospitals	86/275 (31.3)	73/98 (74.5)	43.2 (32.4 to 52.8)	4.28 (2.10 to 8.69)
Academic hospital	58/125 (46.4)	253/302 (83.8)	37.4 (27.5 to 46.7)	3.27 (1.87 to 5.72)
Components of nonoptimal prescribing throughout antimicrobial therapy course				
Prolonged duration^a^	177/400 (44.2)	37/400 (9.2)	−35.0 (−40.2 to −29.2)	0.17 (0.11 to 0.26)
Treatment for asymptomatic bacteriuria^a^	37/400 (9.2)	10/400 (2.5)	−6.8 (−10.0 to −3.4)	0.31 (0.11 to 0.86)
Nonbacterial upper respiratory tract infection^a^	7/400 (1.7)	1/400 (0.3)	−1.5 (−3.0 to 0)	0.15 (0.03 to 0.86)
Non–guideline-concordant selection^b^	81/400 (20.2)	24/400 (6.0)	−14.3 (−18.8 to −9.6)	0.28 (0.10 to 0.78)
Suboptimal dose^c^	23/400 (5.7)	4/400 (1.0)	−4.8 (−7.3 to −2.2)	0.11 (0.03 to 0.43)
Organism resistant to antimicrobial agent^b^	8/400 (2.0)	2/400 (0.5)	−1.5 (−3.2 to 0.2)	0.37 (0.07 to 2.09)
Duration too short^c^	6/400 (1.5)	6/400 (1.5)	0 (−1.8 to 1.8)	0.63 (0.10 to 4.11)

Abbreviations: GEE, generalized estimating equation; OR, odds ratio.

^a^ Indicates unnecessary subcategory for nonoptimal therapy (eMethods in the Supplement), including antimicrobial days beyond indicated duration of therapy, asymptomatic bacteriuria and other noninfectious syndromes, viral respiratory tract infection without bacterial coinfection, and redundant antimicrobial coverage.

^b^ Indicates inappropriate subcategory for nonoptimal therapy (eMethods in the Supplement), including antimicrobial days for an established bacterial infection in which the pathogen is resistant to therapy and antimicrobial selection that is not concordant with institutional guidelines.

^c^ Indicates suboptimal subcategory for nonoptimal therapy (eMethods in the Supplement), including use of an excessively broad-spectrum antimicrobial when a preferred or first-line agent is not contraindicated, dose is too high or too low for kidney function, and duration of therapy is shorter than indicated.

**Table 4.** Patient Outcomes

Outcome	Patient group, No. (%)		Absolute difference, % (95% CI)	Time-adjusted GEE OR (95% CI)
	Preintervention (n = 400)	Postintervention (n = 400)		
30-d Mortality	3 (0.7)	6 (1.5)	0.8 (−0.9 to 2.4)	0.80 (0.09 to 7.18)
90-d Mortality	12 (3.0)	11 (2.7)	−0.2 (−2.7 to 2.2)	0.78 (0.36 to 1.71)
30-d Readmission	77 (19.3)	81 (20.3)	1.0 (−4.5 to 6.5)	0.77 (0.60 to 0.98)
Infection related	33 (8.3)	21 (5.3)	−3.0 (−6.5 to 0.5)	0.48 (0.28 to 0.81)
30-d Unplanned office or emergency department visit	105 (26.3)	109 (27.3)	1.0 (−5.1 to 7.1)	0.59 (0.37 to 0.94)
No clinical resolution^a^	50 (16.5)	34 (12.4)	−4.1 (−9.8 to 1.6)	0.91 (0.63 to 1.30)
Any adverse drug event	78 (19.5)	53 (13.3)	−6.3 (−11.4 to −1.0)	1.09 (0.57 to 2.06)
Severe adverse drug event	36 (9.0)	13 (3.2)	−5.7 (−9.1 to −2.4)	0.40 (0.18 to 0.88)
*Clostridioides difficile* infection	7 (1.7)	2 (0.5)	−1.2 (−2.8 to 0.4)	0.64 (0.11 to 3.64)
MDRO at 90 d	28 (7.0)	10 (2.5)	−4.5 (−7.6 to −1.6)	0.32 (0.15 to 0.71)

Abbreviations: GEE, generalized estimating equation; MDRO, multidrug-resistant organism; OR, odds ratio.

^a^ Includes 303 patients in the preintervention group and 275 in the postintervention group.

After controlling for service team, study month, and other confounders (Table 5), the TOC intervention remained independently associated with the primary outcome, with patients in the postintervention period having nearly 4 times greater odds of being prescribed an optimal antimicrobial regimen at discharge (time-adjusted GEE OR, 3.77 [95% CI, 2.32-6.15]). Length of stay (time-adjusted GEE OR, 0.89 [95% CI, 0.83-0.96]), indications for urinary tract infection (time-adjusted GEE OR, 0.59 [95% CI, 0.44-0.79]), and care at a community hospital (time-adjusted GEE OR, 0.49 [95% CI, 0.38-0.64]) were associated with a lower likelihood of receiving an optimal regimen at discharge.

**Table 5.** Assessment of Covariates and Optimized Discharge Prescription in Univariate and Multivariable Models

Covariate	Optimized discharge prescription^a^		OR (95% CI)^b^	*P* value	Time-adjusted GEE OR (95% CI)^c^	GEE *P* value^c^
	Yes	No				
Overall	470/800 (58.7)	330/800 (41.3)	NA	NA	NA	NA
Postintervention period	326/400 (81.5)	74/400 (18.5)	6.63 (4.45 to 9.86)	<.001	3.77 (2.32 to 6.15)	<.001
Age, mean (SD), y	64.7 (17.6)	69.7 (16.5)	0.83 (0.76 to 0.90)	<.001	0.93 (0.82 to 1.04)	.18
Women	262/441 (59.4)	179/441 (40.6)	0.94 (0.70 to 1.27)	.71	Not tested	NA
Length of stay, median (IQR), d	3 (2-4)	4 (2-5)	0.86 (0.80 to 0.92)	<.001	0.89 (0.83 to 0.96)	.001
Study month	NA	NA	1.25 (1.19 to 1.31)	<.001	1.11 (1.04 to 1.19)	.003
Charlson Comorbidity Index score, median (IQR)	2 (1-3)	2 (1-4)	0.95 (0.88 to 1.03)	.22	Not tested	NA
≥1 MDRO risk factor	251/426 (58.9)	175/426 (41.1)	1.11 (0.83 to 1.50)	.48	Not tested	NA
Community hospital	159/373 (42.6)	214/373 (57.4)	0.23 (0.17 to 0.32)	<.001	0.49 (0.38 to 0.64)	<.001
Urinary source	96/203 (47.3)	107/203 (52.7)	0.58 (0.41 to 0.81)	.002	0.59 (0.44 to 0.79)	<.001
≥2 SIRS criteria on day 3	21/40 (52.5)	19/40 (47.5)	0.79 (0.40 to 1.54)	.49	Not tested	NA
Empirical intravenous antimicrobial	370/650 (56.9)	280/650 (43.1)	0.69 (0.47 to 1.02)	.06	0.76 (0.54 to 1.06)	.11
Dementia	33/71 (46.5)	38/71 (53.5)	0.66 (0.40 to 1.11)	.12	Not tested	NA
Absence of microbiologic or diagnostic data	95/143 (66.4)	48/143 (33.6)	1.19 (0.80 to 1.78)	.39	Not tested	NA

Abbreviations: GEE, generalized estimating equation; MDRO, multidrug-resistant organism; NA, not applicable; OR, odds ratio; SIRS, systemic inflammatory response syndrome.

^a^ Unless otherwise indicated, data are expressed as number/total number (%) of patients.

^b^ Calculated using standard logistic regression. Covariates with an OR greater than 1.00 are associated with an optimized discharge prescription.

^c^ Calculated using multivariable logistic regression. Covariates with an OR greater than 1.00 are associated with an optimized discharge prescription.

## Discussion

In this quality improvement study, the implementation of a pharmacist-led discharge stewardship intervention was associated with improved antimicrobial prescribing in this health system–wide TOC intervention. This outcome was further associated with a reduction in antimicrobial-associated harms, which are common after inappropriate, suboptimal, and/or unnecessary antimicrobial prescribing at hospital discharge.^17,21^ Clinicians face multiple, complex decisions during TOC such as patient placement, costs, education, follow-up, new medications, etc. Pharmacists are crucial team members in leading the supportive antimicrobial reviews (eg, determining antimicrobial days), multidisciplinary discussions, addressing medication access barriers, and facilitating antimicrobial orders into discharge queues for cosigning by the prescriber.

The Centers for Disease Control and Prevention has highlighted the importance of antimicrobial stewardship in the TOC setting in the 2019 core elements of hospital antibiotic stewardship as an opportunity to improve prescribing at hospital discharge.^22,23^ In a multicenter cohort study assessing antimicrobial orders for respiratory and urinary infections from 21 825 discharged patients, Vaughn et al^3^ classified 49% of prescriptions as overuse. These findings were largely owing to excessive durations of therapy and were similar to those in our preintervention group prescribing frequency of prolonged antimicrobial duration at discharge (44.3%). Similar results were observed in a multicenter, cross-sectional study^24^ assessing antimicrobial appropriateness in hospitalized adults. Using objective antimicrobial quality assessment algorithms, more than 75% of antimicrobial courses for community-acquired pneumonia and urinary tract infection were considered unsupported generally because of long duration and incorrect selection of antimicrobial therapy and use for treatment of asymptomatic bacteriuria.^24^

Rigorous education and implementation planning largely contribute to the success of an intervention. Investment from all key partners from medicine, pharmacy, and nursing groups promotes ownership, responsibility, and accountability. Six months of upfront effort was invested from the investigator team to develop the design, feedback structure, training, and education required to implement the intervention across 5 hospitals. Using routine monitoring of protocol adherence, we were able to provide transparency of challenges and successes related to intervention progress via meetings, monthly electronic updates, benchmarking, and positive feedback cases. The additional dedicated time to new interventions in the pharmacist workload varied for each site depending on the population, communication model, and total volume of patients cared for on each shift. A mean of 1 to 3 patients were discharged with a prescription for oral antimicrobials each day from any given service team. In addition to audit and feedback, the pharmacy and antimicrobial stewardship personnel’s active role in executing the intervention via electronic medication entry into the discharge queue was crucial in antimicrobial optimization (eTable 4 in the Supplement). Sustained growth and success of the model was maintained in both academic and community service teams across each quarter during active intervention (eFigure 3 in the Supplement). Replication of similar TOC models may require a foundation of pharmacy leadership, engagement, and clinical expertise, which are resources that are not available to all health care facilities. Zampino et al^8^ observed similar improvement in overall appropriateness of antimicrobial therapy during TOC at an academic hospital after audit and feedback by an antimicrobial stewardship pharmacist for discharge prescriptions from medicine/surgical wards. An interesting finding from our study was that patients discharged from community centers had lower odds of being prescribed an optimal antimicrobial regimen at discharge. These lower odds could be owing to availability of staff in community settings or associated with the study design by having fewer patients in the intervention arm in community hospitals (Table 1).

The synergistic relationships between prescriber and pharmacist in antimicrobial stewardship programs facilitate better care and services.^25^ Medication reconciliation, preauthorization of therapies, and facilitating appropriate diagnostic testing are a few of many examples in which these collaborations have proven beneficial.^11^ Specifically, during TOC, Chavada et al^26^ found large discrepancies between guideline recommendations and the actual antimicrobial discharge prescription. With regard to antimicrobial choice, dose, frequency, and duration, patients who had received an intervention by the antimicrobial stewardship team were more likely to have appropriate therapy.^26^ Yogo et al^9^ used audit and feedback on discharge prescriptions to transition patients with respiratory, skin, urinary, and gastrointestinal tract infections to an optimized antimicrobial selection and duration. Staff pharmacists were trained to conduct this review in real time for patients being discharged from the hospital; duration of therapy after hospital discharge was reduced by a day and prescribing preferences shifted away from fluoroquinolones.^9^ However, appropriateness of the discharge prescription and other clinical end points were unchanged, and among 918 patients prescribed an oral antimicrobial at discharge during the study period, a prescriber was contacted only about 10% of the time.^9^ Our ability to capture more patients in the protocol was likely an important component for improving patient safety.

The intervention was not directly associated with clinical resolution, although we observed an association with reduced severe antimicrobial-related ADEs. Interestingly, no difference in readmissions was observed in the unadjusted analysis; however, after controlling for service team and study month, patients in the postintervention period had a lower risk of 30-day readmission and infection-related readmission. Readmission is a highly confounded outcome, and results should be interpreted with caution. In a large prospective national study, several thousand patients were assigned to receive a variety of interventions related to TOC, which reduced readmissions.^27^ This finding was driven by hospital-based TOC interventions that included but were not limited to medication reconciliation, identification of high-risk patients, and promotion of trust in the hospital. The hospital-based actions were not specific to patients prescribed antimicrobials, although this population should be a high-risk group given the number of comorbid conditions and readmissions associated with infections. In our study, most antimicrobial optimization from the intervention was related to selecting shorter durations and more targeted therapy, which coincides with the ADE frequency before and after the intervention. Each excess antimicrobial day has been associated with 5% greater odds of developing an ADE.^21^ We also found that fewer than 1% (n = 5) of patients had readmissions potentially related to ADEs. Similarly, Vaughn et al^3^ concluded that antimicrobial overuse after discharge was not associated with readmissions, mortality, or patient-reported ADEs; however, incidence of new multidrug-resistant organisms was not assessed.

### Limitations

There are inherent limitations in the nonrandomized design of this study, including biases due to maturation and Hawthorne effect and regression to the mean. Using a nonrandomized stepped-wedge design with multiple observation periods and points of intervention, we were able to better mitigate biases. Notably, the frequency of protocol adherence (63%, defined by the presence of medical record documentation of antimicrobial plan and guideline recommendation by discharge) was surpassed by the frequency of optimal discharge-antimicrobial prescribing (81.5%) in the postintervention group. This occurrence is likely attributable to maturation and the Hawthorne effect given that clinicians became aware of the intervention. We also found differences in diagnoses and race between the preintervention and postintervention groups, likely due to the stepped-wedge intervention rollout order, subsequently leading to imbalances in the numbers of patients from community hospitals. In addition, the process of collaborative rounds for service teams in academic centers was modified to include antimicrobial TOC discussions. Communication models differed between hospitals for paging, telephone calls, and rounding on service teams. There were no other major concurrent health system–wide interventions related to antimicrobial stewardship and/or TOC during the study periods.

## Conclusions

The findings of this quality improvement study suggest that leveraging resources to provide additional review and intervention on antimicrobial discharge therapies may lead to improvements in the quality and safety of antimicrobial prescriptions. Using pharmacists to reinforce institutional protocols, we were able to successfully target and modify the following areas of antimicrobial optimization: minimization of unnecessary antimicrobial days from prolonged durations and patients without infections; avoidance of therapies that are excessively broad, not concordant with local guidance, or targeted toward pathogens that are not susceptible to the antimicrobial; and transitioning from intravenous agents to accessible and affordable oral options as soon as possible. Health care systems seeking to improve quality of prescribing and safety for patients with common infections should consider adopting antimicrobial stewardship interventions at TOC.

## Notes

Supplement. **eMethods.** Definitions **eFigure 1.** Institutional Guidelines for Antimicrobial Selection and Duration of Therapies at the Time of Intervention Implementation **eFigure 2.** Nonrandomized Stepped-Wedge Diagram **eFigure 3.** Proportion of Optimal Discharge Prescribing by Quarter **eTable 1.** Expanded Demographics and Patient Characteristics **eTable 2.** Expanded Patient Outcomes **eTable 3.** Differences in Antimicrobial Length of Therapy (Mean Days) **eTable 4.** Work Flow of Intervention for Antimicrobial Stewardship and/or Clinical Pharmacist
