# Supplementary material for: Pharmacist-Driven Transitions of Care Practice Model for Prescribing Oral Antimicrobials at Hospital Discharge
Source: JAMA Netw Open. 2022 May 10;5(5):e2211331. doi: 10.1001/jamanetworkopen.2022.11331 (PMC9092199; doi:10.1001/jamanetworkopen.2022.11331)
Supplement: Supplement. — eMethods. Definitions eFigure 1. Institutional Guidelines for Antimicrobial Selection and Duration of Therapies at the Time of Intervention Implementation eFigure 2. Nonrandomized Stepped-Wedge Diagram eFigure 3. Proportion of Optimal Discharge Prescribing by Quarter eTable 1. Expanded Demographics and Patient Characteristics eTable 2. Expanded Patient Outcomes eTable 3. Differences in Antimicrobial Length of Therapy (Mean Days) eTable 4. Work Flow of Intervention for Antimicrobial Stewardship and/or Clinical Pharmacist [file jamanetwopen-e2211331-s001.pdf]

Supplementary Online Content

Mercuro NJ, Medler CJ, Kenney RM, et al. Pharmacist-driven transitions of care practice model for prescribing oral antimicrobials at hospital discharge. *JAMA Netw Open*. 2022;5(5):e2211331. doi:10.1001/jamanetworkopen.2022.11331

eMethods. Definitions
eFigure 1. Institutional Guidelines for Antimicrobial Selection and Duration of Therapies at the Time of Intervention Implementation
eFigure 2. Nonrandomized Stepped-Wedge Diagram
eFigure 3. Proportion of Optimal Discharge Prescribing by Quarter
eTable 1. Expanded Demographics and Patient Characteristics
eTable 2. Expanded Patient Outcomes
eTable 3. Differences in Antimicrobial Length of Therapy (Mean Days)
eTable 4. Work Flow of Intervention for Antimicrobial Stewardship and/or Clinical Pharmacist

This supplementary material has been provided by the authors to give readers additional information about their work.

© 2022 Mercuro NJ et al. *JAMA Network Open*.

eMethods. Definitions

Definitions

Clinical resolution

- Complete resolution of signs and symptoms (fever, leukocytosis, hypotension, respiratory failure, and localizing symptoms) such that no further antimicrobial therapy is required; assessed at end of treatment and follow up

Adverse effects

- Mild/Moderate
  - Rash, transaminitis, altered mental status, diarrhea, nausea/vomiting, prolonged QTc without cardiac event, acute renal risk, electrolyte abnormality without cardiac/neurologic event while on therapy
- Serious
  - *Clostridiodes difficile* (CDI) within 90 days of discharge, development of multi-drug resistant organism (MDRO) within 90 days of discharge, anaphylaxis, rhabdomyolysis, acute renal injury/failure, seizure, drug-induced thrombocytopenia/neutropenia/anemia, Steven-Johnson-Syndrome/Toxic epidermal necrosis while on therapy

Antimicrobial optimization- an optimal antimicrobial regimen does not have any of the following components:

- Unnecessary
  - For noninfectious syndromes (e.g., asymptomatic bacteriuria), nonbacterial infections (e.g., viral respiratory tract infection without superimposed pneumonia), days of therapy beyond indicated duration of therapy absent of any clinical reason for lengthened course, use of redundant antimicrobial therapy (e.g., double-anaerobic coverage)
- Inappropriate
  - Use of antimicrobial in setting of established infection to which the pathogen is resistant, or use is not concordant in institutional treatment guidelines (e.g., fluoroquinolone for cystitis when other options available)
- Suboptimal
  - Use of antimicrobials in the setting of established infection that can be improved by drug: (1) selection, (2) route, (3) dose and interval
    - Use of overly broad agent to treat susceptible bacterium within a lesser category antimicrobial if allergies do not preclude its use
    - Use of intravenous (IV) when oral (PO) equivalent with high bioavailability is an option based on IV to PO institutional guideline
    - Dose too high or too low for infection and/or renal function

Target Disease States:

- Intra-Abdominal Infection (IAI):
  - ***Spontaneous bacterial peritonitis*** confirmed by paracentesis and polymorphonuclear leukocytes greater than or equal to 250 cells/cc.
  - Radiographic evidence of ***cholecystitis, cholangitis, appendicitis, diverticulitis,*** or ***abdominal abscess*** following surgical source control
- Urinary Tract Infection (UTI):
  - ***Asymptomatic bacteriuria (ASB)***: Antimicrobial indication for UTI and lack of documentation for urinary symptoms listed below. Appropriate antimicrobial duration for non-pregnant ASB was zero days
  - ***Cystitis***: dysuria, supra-pubic pain, increased urinary frequency, altered mental status without other potential causes, without fever, flank pain, or other suspicious findings of pyelonephritis or complicated UTI
  - ***Pyelonephritis***: ICD code and/orchart documentation at physician discretion

© 2022 Mercuro NJ et al. *JAMA Network Open*.

    - ***Complicated urinary tract infection:*** all other urinary tract infection not meeting the above criteria for urinary tract infection
- Skin/Soft Tissue Infection (SSTI):
    - Includes cellulitis/erysipelas, wound infection, and cutaneous abscesses assigned by ICD code and/or documentation as hospital problem
- Respiratory Tract Infection:
    - ***Community-Acquired Pneumonia (CAP)***: Presentation of pneumonia less than 48 hours from hospital admission, with or without risk factors for multi-drug resistant organisms. Diagnosed by physician, with radiographic evidence.
    - ***Hospital-associated pneumonia (HAP)***: Presentation of pneumonia greater than 48 hours from hospital admission. Diagnosed by physician, with radiographic evidence.
    - ***Respiratory viral infection (RVI)***: Positive testing for a respiratory virus without radiographic evidence of pneumonia OR positive testing, radiographic findings, and procalcitonin <0.25. Appropriate antimicrobial duration for RVI is 0 days and if influenza positive, 5 days of oseltamivir.

Excluded disease states, populations, and organisms:

- Solid organ transplant
- Neutropenia (ANC <500 cells/cc)
- Cystic fibrosis
- HIV with CD4 <200 cells/cc
- Primary immunodeficiencies
- Age <18 years
- Discharge location hospice
- Discharge with intravenous antimicrobials
- Transferred from outside hospital
- Severe/complicated infections
    - Endocarditis
    - Meningitis/Central nervous system
    - Lack of source control
    - Febrile neutropenia
    - Prostatitis/orchitis/epididymitis
    - Osteoarticular infections
    - Diabetic foot infection
    - Necrotizing fasciitis
    - Hidradenitis suppurativa
    - Meningitis, brain abscess
    - Mediastinitis
    - Bloodstream due to fungi, *S. aureus, Enterococci*
    - Fungal pneumonia
    - Empyema, lung abscess
    - *Actinomyces* infection
    - *Aspergillus* infection
    - Mycobacterial infections

© 2022 Mercuro NJ et al. *JAMA Network Open*.

eFigure 1. Institutional Guidelines for Antimicrobial Selection and Duration of Therapies at the Time of Intervention Implementation

Institutional Oral Antimicrobial Selection and Duration Guidance*			
Respiratory Tract Infections	Community-acquired pneumonia, with or without risk factors (without microbiologic data)	• Amoxicillin-clavulanic acid 1000/62.5 mg 2 tabs BID + (azithromycin 500 mg daily or doxycycline 100 mg BID)<br>• Cefuroxime 500 mg BID OR cefpodoxime 400 mg BID + (azithromycin 500 mg daily or doxycycline 100 mg BID)<br>• Doxycycline 100 mg BID<br>• Moxifloxacin 400 mg OR levofloxacin 750 mg daily	5 days in patients with prompt clinical response 7-10 days in patients with structural lung disease or delayed response
	Acute exacerbation of COPD (AECOPD)	• Doxycycline 100 mg BID<br>• Azithromycin 500 mg x1 then 250 mg daily	5-7 days
	Hospital acquired pneumonia	• Moxifloxacin 400 mg OR levofloxacin 750 mg daily	7 days w/ prompt clinical response: tailor therapy to microbiologic data
	Influenza	• Oseltamivir 75 mg BID	5 days
Urinary Tract Infections	Uncomplicated UTI/cystitis: Align with organism susceptibility	• Nitrofurantoin (NFT) 100 mg BID<br>• Sulfamethoxazole/trimethoprim (SMT) 1 DS tab BID<br>• Beta-lactam (targeted to organism)<br>• Fosfomycin 3 gm oral sachet (MDRO history only)	• NFT: 5 days<br>• SMT: 3 days<br>• Beta-lactams: 3-7 days<br>• Fosfomycin: 2-3 doses
	Complicated UTI/ pyelonephritis Align with organism susceptibility	• Sulfamethoxazole/trimethoprim (SMT) 1-2 DS tab BID<br>• Ciprofloxacin 500 mg BID<br>• Beta-lactams (targeted to organism)	• SMT: 10-14 days*<br>• Fluoroquinolones: 7 days<br>• Beta-lactams: 10-14 days
	Asymptomatic bacteriuria	• Do not treat if not pregnant, or perioperative prophylaxis	0 days

© 2022 Mercuro NJ et al. *JAMA Network Open*.

Institutional Oral Antimicrobial Selection and Duration Guidance			
Skin Structure Infection	Non-purulent cellulitis	• Cephalexin 500 mg QID, Cefuroxime 500 mg BID<br>• Dicloxacillin 500 mg QID<br>• Clindamycin 300-450 mg TID (severe beta lactam allergy)	5 days with prompt clinical response
	Purulent cellulitis/cutaneous abscess (suspected MRSA)	• Doxycycline 100 mg BID<br>• Sulfamethoxazole/trimethoprim 1-2 DS BID	5 days with prompt clinical response
Intra-abdominal infection	Spontaneous bacterial peritonitis	• Moxifloxacin 400 mg or levofloxacin 750 mg daily	5 days
	Complicated, community acquired intra-abdominal infection **with source control** e.g., appendicitis, cholecystitis, diverticulitis s/p removal of foci	• Moxifloxacin 400 mg daily<br>• Ciprofloxacin 500 mg BID + metronidazole 500 mg BID/TID<br>• Cefuroxime 500 mg BID + metronidazole 500 mg BID<br>• Amoxicillin-clavulanic acid 875/125 mg BID	4-7 days after source control<br>7 days targeted therapy in transient bacteremia after foci removed

*Disclaimer: Guidelines for oral antimicrobial selection and duration were developed at the time of the intervention implementation. Local guidance should be considered with antibiogram and updated literature.

© 2022 Mercuro NJ et al. *JAMA Network Open*.

eFigure 2. Nonrandomized Stepped-Wedge Diagram

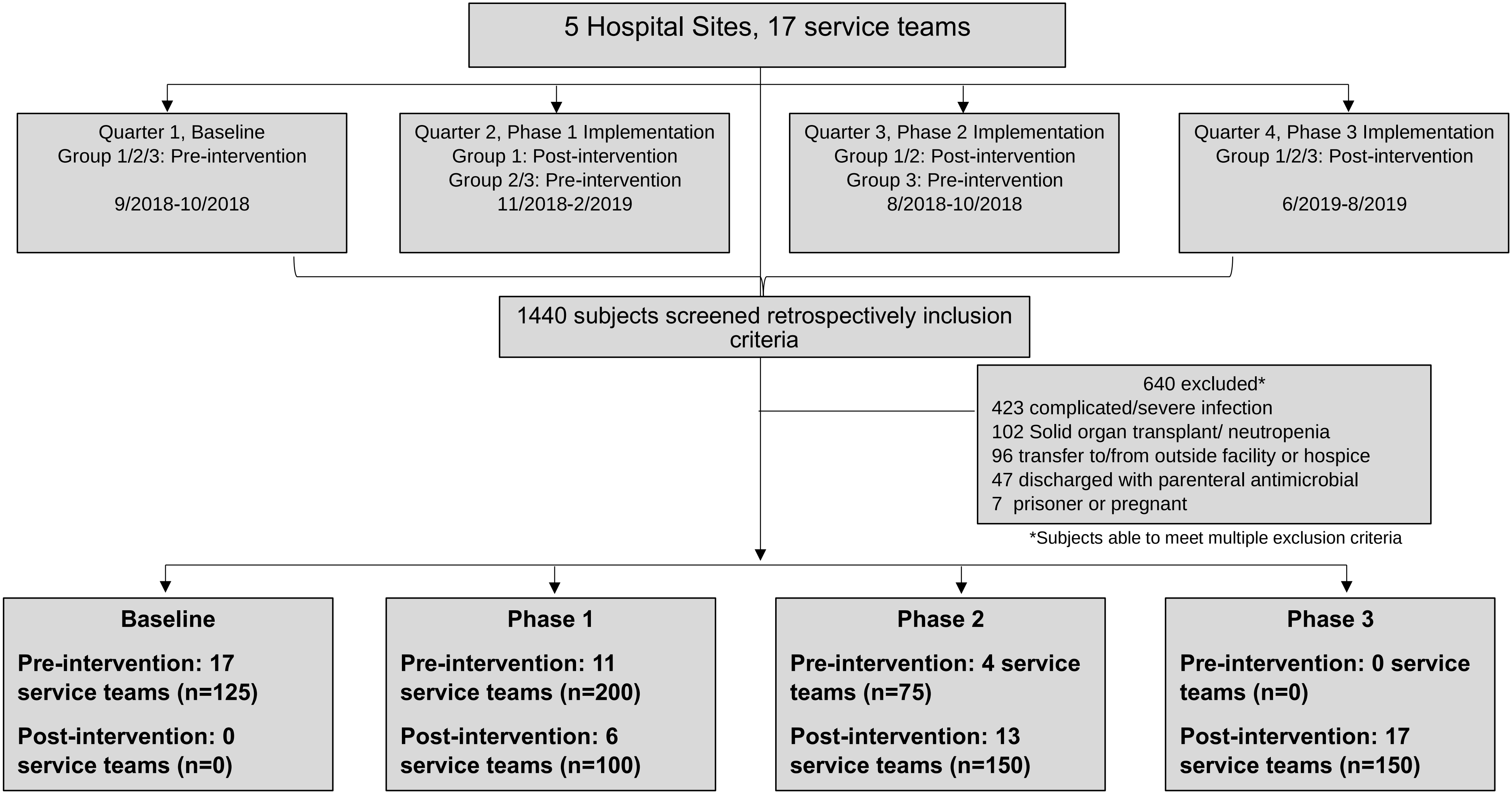

© 2022 Mercuro NJ et al. *JAMA Network Open*.

eFigure 3. Proportion of Optimal Discharge Prescribing by Study Phase and Quarter

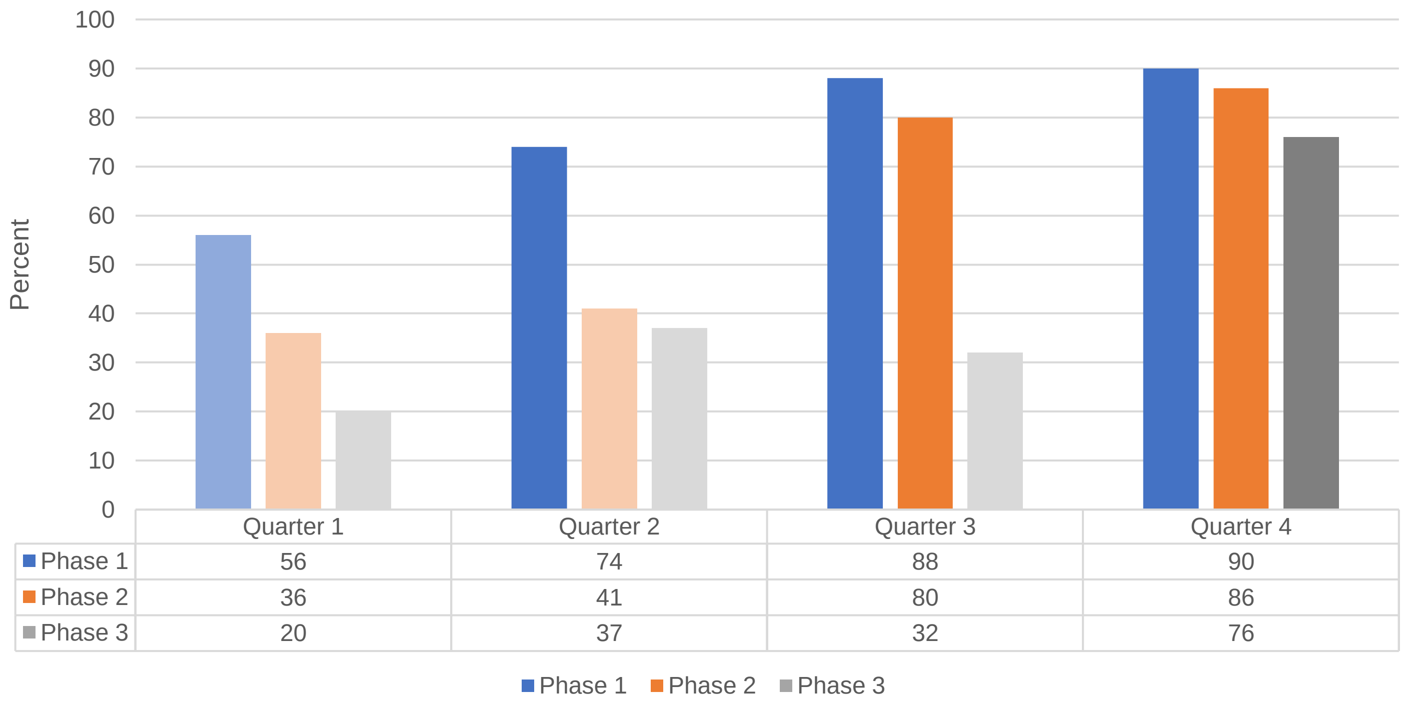

	Quarter 1	Quarter 2	Quarter 3	Quarter 4
Phase 1	56	74	88	90
Phase 2	36	41	80	86
Phase 3	20	37	32	76

Lighter shades denote pre-intervention periods, darker shades denote post-intervention period.
Phase 1 (n=250) consisted of units from academic medical center only, overall mean difference in optimal prescribing between pre- and post-intervention periods +26.2%
Phase 2 (n=275) consisted of units from both academic and community medical centers, overall mean difference in optimal prescribing between pre- and post-intervention periods +43.1%
Phase 3 (n=275) consisted of units from community medical centers, overall mean difference in optimal prescribing between pre- and post-intervention periods +44.4%

© 2022 Mercuro NJ et al. *JAMA Network Open*.

eTable 1. Expanded Demographics and Patient Characteristics

	Overall Pre- (n=400)	Overall Post- (n=400)	Phase 1 Pre- (n=25)	Phase 1 Post- (n=225)	Phase 2 Pre- (n=150)	Phase 2 Post- (n=125)	Phase 3 Pre- (n=225)	Phase 3 Post- (n=50)
Age, years (± SD)	69.0 (17.1)	64.5 (17.2)	55.9 (18.9)	61.7 (17.5)	64.6 (15.7)	66 (16.7)	73.4 (16.4)	73.6 (13.4)
Female sex, n (%)	221 (55.3)	220 (55)	128 (56.9)	11 (44)	81 (54)	69 (55.2)	129 (57.3)	23 (46)
Race, n (%)								
• Black	113 (28.2)	200 (50)	16 (64)	142 (63.1)	71 (47.3)	52 (41.6)	26 (11.6)	6 (12)
• White	259 (64.8)	161 (40.3)	6 (24)	60 (26.7)	65 (43.3)	58 (46.4)	188 (83.6)	43 (86)
• Other/unknown	28 (7)	39 (9.7)	3 (12)	23 (10.2)	14 (8.6)	15 (12)	11 (4.8)	1 (2)
Charlson Score, median (IQR)	2 (1-3)	2 (1-3)	1 (0-3)	1 (1-3)	3 (2-4)	3 (3-5)	4.2 (2.3)	4.1 (3.1)
SIRS ≥2 on day 1, n (%)	116 (29)	134 (33.5)	13 (52)	86 (38.2)	28 (18.7)	37 (29.8)	75 (33.3)	11 (22)
SIRS ≥2 on day 3, n (%)	22 (5.5)	18 (4.5)	1 (4)	11 (4.9)	5 (3.3)	4 (3.2)	16 (7.1)	3 (6)
Length of stay, days (± SD)	3.6 (2.2)	3.3 (2.2)	3.0 (1.5)	3.4 (1.9)	3.5 (2.2)	4.0 (2.3)	3.9 (2.4)	3.7 (3.0)
Any MDRO risk factor, n (%)	216 (54)	210 (52.5)	10 (40)	117 (52)	86 (57.3)	62 (49.6)	120 (53.3)	31 (62)
• Admit in last 90 days	130 (32.5)	117 (29.3)	9 (36)	61 (27.1)	49 (32.7)	38 (30.4)	72 (32)	18 (36)
• Antimicrobial in last 90 days	144 (36)	154 (38.5)	8 (32)	82 (36.4)	55 (36.7)	45 (36)	81 (36)	27 (54)
• Prior MDRO colonization	22 (5.5)	27 (6.8)	2 (8)	13 (5.8)	8 (5.3)	10 (8)	12 (5.3)	4 (8)
• Immunocompromised	7 (1.8)	15 (3.8)	1 (4)	12 (5.3)	1 (0.7)	3 (2.4)	5 (2.2)	0
• Non-ambulatory status	32 (8)	19 (4.8)	2 (8)	14 (6.2)	11 (7.3)	3 (2.4)	19 (8.4)	2 (4)
Pneumonia, n (%)	144 (36)	120 (30)	6 (24)	64 (28.4)	45 (30)	39 (31.2)	93 (41.3)	17 (34)
• CAP without risk factors	108 (27)	92 (23)	3 (12)	48 (21.3)	39 (26)	30 (24)	66 (29.3)	14 (28)
• CAP with risk factors	33 (8.3)	24 (6)	2 (8)	13 (5.8)	6 (4)	8 (6.4)	25 (11.1)	3 (6)
• HAP	3 (0.8)	4 (1)	1 (4)	3 (1.3)	0	1 (0.8)	2 (0.9)	0
AECOPD/ upper respiratory tract infection, n (%)	101 (25.3)	113 (28.2)	2 (8)	64 (28.4)	43 (28.7)	39 (31.2)	56 (24.9)	10 (20)

© 2022 Mercuro NJ et al. *JAMA Network Open*.

	**Overall Pre- (n=400)**	**Overall Post- (n=400)**	**Phase 1 Pre- (n=25)**	**Phase 1 Post- (n=225)**	**Phase 2 Pre- (n=150)**	**Phase 2 Post- (n=125)**	**Phase 3 Pre- (n=225)**	**Phase 3 Post- (n=50)**
Urinary tract infection (UTI)	115 (28.7)	88 (22)	6 (24)	44 (19.6)	42 (28)	32 (25.6)	67 (29.8)	12 (24)
• Pyelonephritis	25 (6.3)	23 (5.8)	3 (12)	12 (5.3)	8 (5.3)	10 (8)	14 (6.2)	1 (2)
• Complicated UTI	46 (11.5)	39 (9.8)	1 (4)	22 (9.8)	16 (10.7)	11 (8.8)	29 (12.9)	6 (12)
• Cystitis	44 (11)	26 (6.5)	2 (8)	10 (4.4)	18 (12)	11 (8.8)	24 (10.7)	5 (10)
Skin/soft tissue infection	53 (13.3)	72 (18)	11 (44)	49 (21.8)	22 (14.7)	13 (10.4)	20 (8.9)	10 (20)
• Purulent	20 (5)	39 (9.8)	6 (24)	31 (13.8)	8 (5.3)	5 (4)	6 (2.7)	3 (6)
• Non-purulent	33 (8.3)	33 (8.3)	5 (20)	18 (8)	14 (9.3)	8 (6.4)	14 (6.2)	7 (14)
Intra-abdominal infection	4 (1)	12 (3)	0 (0)	9 (4)	2 (1.3)	3 (2.4)	2 (0.9)	0
Disposition, n (%)								
• Home/home services	328 (82)	345 (86.3)	21 (84)	193 (85.8)	128 (85.3)	111 (88.8)	179 (79.6)	41 (82)
• Long-term care facility/ skilled nursing facility	69 (17.3)	51 (12.8)	4 (16)	28 (12.4)	22 (14.7)	14 (11.2)	43 (19.1)	9 (18)
• Left against medical advice	3 (0.8)	4 (1)	0 (0)	4 (1.8)	0 (0)	0 (0)	3 (1.3)	0 (0)

AECOPD- acute exacerbation of chronic obstructive pulmonary disorder

© 2022 Mercuro NJ et al. *JAMA Network Open*.

eTable 2. Expanded Patient Outcomes

	Overall Pre- (n=400)	Overall Post- (n=400)	Phase 1 Pre- (n=25)	Phase 1 Post- (n=225)	Phase 2 Pre- (n=150)	Phase 2 Post- (n=125)	Phase 3 Pre- (n=225)	Phase 3 Post- (n=50)
Optimized discharge regimen	144 (36)	326 (81.5)	14 (56)	185 (82.2)	59 (39.3)	103 (82.4)	71 (31.6)	38 (76)
30-day mortality	3 (0.8)	6 (1.5)	0 (0)	3 (1.3)	0 (0)	3 (2.4)	3 (1.3)	0
90-day mortality	12 (3)	11 (2.8)	1 (4)	6 (2.7)	4 (2.7)	4 (3.2)	7 (3.1)	1 (2)
30-day readmission	77 (19.3)	81 (20.3)	4 (16)	38 (16.9)	28 (18.7)	28 (22.4)	45 (20)	15 (30)
• Infection related	33 (8.3)	21 (5.3)	3 (12)	14 (6.2)	10 (6.7)	4 (3.2)	20 (8.9)	3 (6)
Clinical resolution	253/303 (83.5)	241/275 (87.6)	13/17 (76.5)	130/153 (85)	82/96 (85.4)	78/88 (88.6)	158/190 (83.2)	33/34 (97.1)
Any adverse drug event	78 (19.8)	53 (13.6)	8 (32)	32 (14.4)	20 (13.4)	17 (13.9)	50 (22.7)	4 (8.5)
Severe adverse drug event (all)	36 (9)	13 (3.3)	6 (24)	8 (3.6)	8 (5.3)	4 (3.2)	22 (9.8)	1 (2)
• *C. difficile*	7 (1.8)	2 (0.5)	3 (12)	1 (0.4)	0	0	4 (1.8)	1 (2)
• MDRO[a] at 90 days	28 (7)	10 (2.5)	3 (12)	6 (2.7)	7 (4.7)	4 (3.2)	18 (8)	0

[a]Multi-drug resistant organism
Phases 1, 2, 3 represent three groups of hospital service teams, implemented in accordance to the stepped wedge design

© 2022 Mercuro NJ et al. *JAMA Network Open*.

eTable 3. Differences in Antimicrobial Length of Therapy (Mean Days)

	Unadjusted				Time Adjusted Estimates			
	Pre-	Post-	Absolute % Difference (95% CI)	P-value	Pre-	Post-	Absolute % Difference (95% CI)	P-value
Total (n=800)	8.9	7.4	-1.5 (-1.9 to -1.0)	<0.001	8.7	7.6	-1.1 (-1.7 to -0.6)	<0.001
Outpatient (n=800)	5.5	4.6	-0.9 (-1.3 to -0.6)	<0.001	5.3	4.8	-0.5 (-1.0 to 0.0)	0.05
Total, Respiratory infection (n=455)	8.3	6.4	-1.9 (-2.3 to -1.4)	<0.001	8.2	6.5	-1.8 (-2.3 to -1.2)	<0.001
Total, Urinary tract infection (n=203)	9.7	8.1	-1.6 (-2.6 to -0.6)	0.002	9.4	8.5	-0.9 (-2.2 to 0.4)	0.16
Total, Skin/soft tissue infection (n=125)	9.7	9.8	0.0 (-1.2 to 1.3)	0.94	9.5	10.0	+0.5 (-1.2 to 2.2)	0.58

© 2022 Mercuro NJ et al. *JAMA Network Open*.

eTable 4. Work Flow of Intervention for Antimicrobial Stewardship and/or Clinical Pharmacist

	**Base TOC model**	**Modifications made to optimize process for individual practice setting**
**Identifying patients approaching discharge**	• Electronic medical record included datapoints for anticipated discharge date<br>• Patients approaching discharge on antimicrobials discussed on collaborative rounds	• Daily reports obtained from case managers<br>• Nursing managers contacted on individual units for upcoming discharges<br>• Outpatient pharmacy contacted inpatient pharmacy team for new prescriptions for oral antimicrobials on patients who do not have a documented antibiotic discharge plan
**Patient therapy assessments**	• Institutional guidelines applied<br>• Prospective audit and feedback of patients with active antimicrobials conducted weekdays daily 7am-4pm<br>• Clinical assessments conducted on inpatient and discharge antimicrobial therapy	• Weekday assessments conducted by either antimicrobial stewardship or clinical pharmacist, depending on resources and care model<br>• Weekend assessments conducted by antimicrobial stewardship, or not performed, depending on care model
**Communication**	• Individual cases discussed during collaborative rounds<br>• Therapy plan documented in progress notes after confirmation with team	• Paging or phone call communication in non-rounding models
**Discharge order entry**	• Orders entered into discharge queue for cosigning by primary team (prior to discharge) with stop date<br>• Orders modified in discharge queue during rounds, if needed	• Paging or phone call to prescriber to review/ modify the discharge prescription, if needed, when discharge orders have already been entered. Timely discharge of the patient was prioritized and the ability to conduct the intervention was designed to not interfere with or delay the discharge process

© 2022 Mercuro NJ et al. *JAMA Network Open*.
